# Evaluation of Single and Combined Temozolomide and Doxorubicin Treatment Responses in Low- and High-Grade Glioma In Vitro

**DOI:** 10.7759/cureus.66928

**Published:** 2024-08-15

**Authors:** Georgiana Adeline Staicu, Ligia G Tataranu, Daniela Elise Tache, Stefana Oana Popescu, Stefan Alexandru Artene, Suzana Danoiu, Veronica Sfredel, Edmond Nicolae Barcan, Stefania Carina Baloi, Anica Dricu

**Affiliations:** 1 Department of Biochemistry, University of Medicine and Pharmacy of Craiova, Craiova, ROU; 2 Department of Neurosurgery, Bagdasar-Arseni Clinical Emergency Hospital, Bucharest, ROU; 3 Department of Neurosurgery, Carol Davila University of Medicine and Pharmacy, Bucharest, ROU; 4 Department of Physiology, University of Medicine and Pharmacy of Craiova, Craiova, ROU

**Keywords:** cytotoxicity, temozolomide, doxorubicin, astrocytoma, glioblastoma

## Abstract

Background: Astrocytoma, the most common type of glioma, can histologically be low or high grade. Treatment recommendations for astrocytic tumors are based on the histopathological and molecular phenotype. For grade 2 astrocytoma, the combination of radiotherapy and adjuvant chemotherapy with procarbazine, lomustine, and vincristine (PCV) is better than radiotherapy alone. Temozolomide (TMZ) is being increasingly recognized as a replacement for PCV in brain tumor therapy, due to the lower myelotoxicity. TMZ is currently a well-established first-line treatment for grade 3 astrocytoma, grade 4 astrocytoma, and glioblastoma and it is also sporadically used for grade 2 astrocytoma. However, TMZ faces multiple challenges such as adverse effects and drug resistance.

Methods: In this study, we compared the cytotoxic effect induced by TMZ and doxorubicin (DOXO), alone and in combination, on a low-grade astrocytoma cell line (AC1B) and a high-grade glioma cell line (GB1B).

Results: We found that TMZ and DOXO, each produced a cytotoxic effect in monotherapy. GB1B cell line was more sensitive to the treatment than AC1B cells, at a 7- and 10-day exposure to the DOXO. However, when the duration of the treatment was extended to 14 days, GB1B cells became more resistant to DOXO treatment, compared to AC1B cells. Regarding the treatment with TMZ, GB1B exhibited greater resistance to TMZ compared to AC1B, across all studied intervals and the resistance to treatment of GB1B increased with longer exposure time. However, in combined therapy, the drugs did not exert a synergistic effect on any astrocytic cell line.

Conclusions: The current data suggest that both TMZ and DOXO exhibit efficient therapeutic effects on low- and high-grade glioma cells. However, no synergistic effect was observed for combined therapy.

## Introduction

Glioma is a common type of central nervous system (CNS) tumor, originating from the glial cells, including astrocytes, ependymal cells, and oligodendrocytes. According to their aggressiveness, they can be subdivided into low-grade and high-grade gliomas. From a histopathological point of view, diffuse gliomas are categorized as astrocytic, oligodendroglial, and oligoastrocytic, to which features like nuclear atypia, necrosis, microvascular proliferation (MVP), and proliferative index are added to further grade the tumor. Nuclear atypia may be present in lower-grade tumors, while the addition of increased mitotic activity and MVP increases the grade. Generally, necrosis and MVP in a “glomeruloid” fashion, along with increased mitotic activity, may be associated with glioblastoma or higher-grade glioma. Other features, such as calcifications and branching capillaries, may be associated with oligodendroglial tumors. To fully characterize glial tumors, additional tests such as immunohistochemistry and molecular analysis are mandatory. Each grade carries a different prognosis and different treatment options [1].

The most common type of glioma in both adult and pediatric populations is astrocytoma, which are low-grade glioma originating from astrocytes. There are four grades of astrocytoma. Grade 1 or non-infiltrating astrocytomas (slow-growing tumors) do not typically expand into the surrounding tissue. Low-grade astrocytomas (grade 2) are tumors that can infiltrate surrounding tissues, usually with slow development but with quicker growth over time. Grade 3 previously described as anaplastic astrocytoma grows faster than grade 2 astrocytoma. The most aggressive and fastest-growing types of all CNS tumors are grade 4 astrocytomas and glioblastomas [2].

Most astrocytomas have an unknown cause. Certain types of cancer may be brought on by genetic and immunologic abnormalities, environmental factors (such as exposure to certain chemicals, UV rays, and ionizing radiation), nutrition, stress, and other causes.

Grade 1 astrocytoma more frequently affects children and adolescents, while grade 2 astrocytoma is typically diagnosed in adults aged 20 to 60. Grade 3 astrocytoma most frequently affects adults between the ages of 30 and 60, while grade 4 astrocytoma has been depicted generally in adults between the ages of 50 and 80. The previously known entity “anaplastic astrocytoma” was described to be more common in men, and accounted for 4% of all brain tumors [3].

Most low-grade astrocytomas can be cured, but the outlook is less encouraging for patients with a high-grade tumor. For grade 2 astrocytoma, the average survival time is 6-8 years after surgery, and more than 40% of patients live for more than 10 years. However, just 25% of patients with grade 4 astrocytoma survive beyond one year mark [4].

Low-grade gliomas include astrocytomas and oligodendrogliomas. Standard of care is represented by maximal safe surgical resection. When removing a tumor from healthy brain tissue proves to be too challenging or when the tumor cannot be reached, radiation therapy (RT) is used. RT is used in people over 10 years of age, with a tumor that cannot be surgically removed. Chemotherapy may be used in place of RT if the patient is younger than 10 years old because RT can hinder young children's brain growth and development [5]. According to the recently published data, it is now recommended that patients at high risk should undergo both radiation and chemotherapy following surgery. However, they do not address the management of low-grade glioma patients in the era of genomic medicine [6].

Glioblastoma is a high-grade glioma and the most aggressive of all malignant brain tumors originating from glial cells. There is no known precise cause of glioblastoma. Some are associated with rare genetic syndromes such as Li-Fraumeni and neurofibromatosis, but most cases are sporadic, without being genetically transmitted. Glioblastomas used to be characterized in two main types, based on their evolution: primary glioblastoma (appears from the very beginning as a malignant, aggressive tumor, evolves rapidly and responds poorly to radiotherapy and chemotherapy) and secondary glioblastoma (initially appears as a lower grade tumor, evolves asymptomatic for a long period of time and transforms into grade 4 glioblastoma). The response of the previously known entity “secondary glioblastoma” to radiotherapy and chemotherapy is much better than in primary glioblastoma [3,7].

Chemotherapy and RT resistance is very common in brain tumor patients. The notion of chemotherapy has been replaced by targeted therapy, as a result of the emergence of new anti-cancer medications. These novel medications produce greater and more targeted cell death, but their effects are also transient since cancer cells quickly develop treatment resistance [7]. Moreover, another obstacle to anti-astrocytoma treatment is the lack of active components that can pass through the blood-brain barrier (BBB) and the blood-brain tumor barrier (BBTB) to have good bioavailability in the targeted tissue [8].

Over time, the treatment of WHO tumor grade 2 or grade 3 astrocytomas has traditionally involved various therapeutic interventions, making it difficult for medical professionals, patients, and caregivers to choose the ideal timing and strategy for determining appropriate therapy. It is crucial to thoroughly evaluate the potential side effects in cancer management. Initial safe limit resection is the standard of care for treating astrocytomas. Generally, adjuvant RT after surgical resection is started in high-risk patients, followed by lomustine, procarbazine, and vincristine treatment for patients with astrocytoma WHO grade 2 and maintenance temozolomide (TMZ) treatment for patients with WHO grade 3 astrocytomas [9]. It is important to mention that the use of chemotherapy medications in monotherapy is still being studied with limited data available. In high-risk astrocytomas, monotherapy with TMZ has been linked to decreased progression-free survival (PFS) compared to radiotherapy alone [10].

Glioblastoma (a grade 4 tumor) is still incurable and new treatments such as molecular therapy, personalized therapy, nanomedicine, etc., are constantly developed and studied [11,12].

However, TMZ remains the first-line drug used in the treatment of both glioblastomas and astrocytomas. It has a lipophilic nature (alkylating agent from the imidazotetrazine class) that permits its passage through the BBB. It may be administered orally or intravenously [13]. In fact, TMZ is a prodrug of 3-methyl-(triazen-1-yl)imidazole-4-carboxamide (MITC). Its mechanism of action is based on the addition of a methyl group to the purine bases of DNA at various levels: guanine N7, adenine N3, and residues of guanine O6 [14]. The antitumor effectiveness mainly results from the 06-methylguanine formation, which pairs incorrectly with thymine, a mutagenic, carcinogenic, and toxic lesion. This mutation gets to be repeatedly repaired through DNA mismatch repair (MMR), and eventually, the process induces DNA damage finalized by cell cycle arrest (G2/M), cell death, and apoptosis. Therefore, for TMZ to have a pharmacological effect, it requires a functional MMR mechanism [13]. Tumor progression occurs in approximately 90% of patients due to the development of TMZ resistance, resulting in no response to the subsequent chemotherapy cycle [15]. Currently, overcoming the myelosuppressive toxicity induced by TMZ while maintaining its effectiveness represents a challenge [16]. A valuable strategy for dealing with the challenging therapeutic control of the disease is the co-administration of multiple active therapeutic drugs or a combination of other strategies. Studies conducted both in vitro and in vivo showed that combination therapeutic approaches could provide significant benefits, such as achieving a mutually beneficial impact with the chance to reduce the risk of side effects and also decreasing the risk of developing resistance [17-19].

Trials involving TMZ and other drugs like interferon-beta and Zotiraciclib have been conducted.

The induction of O6-methylguanine-DNA methyltransferase in tumors is considered to be one of the main causes of resistance to TMZ. Studies have demonstrated that interferon-beta suppresses O6-methylguanine-DNA methyltransferase in experimental glioma models, among other drugs. Recent findings reported that patients diagnosed with TMZ-refractory anaplastic astrocytoma have been successfully treated with a combination of TMZ and interferon-beta [20].

Another studied combination therapy was TMZ with Zotiraciclib, a pyrimidine-based multi-kinase inhibitor exhibiting inhibitory effects on cyclin-dependent kinases. Wu and colleagues reported that Zotiraciclib combined with TMZ is safe in recurrent high-grade astrocytoma patients. Zotiraciclib-induced neutropenia has been transient [21].

Doxorubicin (DOXO) is an anthracycline antineoplastic drug, characterized by antimitotic and cytotoxic activity through a number of mechanisms of action. DOXO forms complexes with DNA and inhibits topoisomerase II action by stabilizing the DNA-topoisomerase II complex by intercalation between base pairs [22].

Based on its mechanism of action, DOXO treatment produces regression in disseminated malignant neoplasms: acute myeloblastic leukemia, acute lymphoblastic leukemia, neuroblastoma, bone and soft tissue sarcomas, breast and ovarian cancer, thyroid and gastric carcinoma. As with other chemotherapeutic drugs, DOXO expresses a variety of adverse effects, including nephrotoxicity, cardiotoxicity, and bone marrow suppression [23]. Nonetheless, DOXO presents limited BBB penetration, thus it is not included in the standard of care for brain tumors [24].

The use of drug combinations has the potential to increase the efficacy of a treatment. We have previously determined the combined effects of DOXO and TMZ in cultured glioblastoma cells and demonstrated that both medications produced cytotoxic effects on the GB2B cell line [25]. Therefore, in the current study, we extended the research to determine the effects of the treatment on astrocytic tumor cells. We also chose two drugs that belong to different classes: TMZ, a small lipophilic alkylating agent and DOXO, an anthracycline antibiotic. The cytotoxic effects of both single and combined treatment were investigated in vitro.

## Materials and methods

Cell lines and culture conditions

The study was carried out using two glioma cell lines: a grade 2 astrocytoma cell line (AC1B) and a glioblastoma cell line (GB1B). Both low- and high-grade glioma cells were obtained from patients diagnosed at Bagdasar-Arseni Clinical Emergency Hospital, Bucharest, Romania. AC1B and GB1B cell lines were established according to standard procedures [26].

Cell cultures were grown in minimum essential medium (MEM) supplemented with 10% fetal bovine serum (FBS), 2mM of glutamine, 100IU/mL streptomycin, and 100IU/mL penicillin in tissue cultivation flasks, kept in a humidified incubator at a temperature of 37°C and 5% CO_2_.

Cell treatment

Cell seeding was performed in 96-well plates (0.5-1-3×10^3 ^cells/well). The study was conducted on both cell lines under the same experimental conditions, as follows: one control consisting of either astrocytoma or glioblastoma cells was treated with diluents, the second group of each cell line was treated with two concentrations of DOXO (10μM and 100μM), another group was treated with two concentrations of TMZ (10μM and 100μM) and the fourth group of each cell line was treated with a low dose amalgam of both drugs (DOXO 10μM and TMZ 10μM). The cytotoxic activity was measured at three different time intervals (7 days, 10 days, and 14 days). The anticancer medications were introduced every two days. Each experiment was conducted three times under the same conditions.

Liquid management

By utilizing an EpMotion 5070 instrument (Eppendorf, Hamburg, Germany), the samples containing the cell lines, chemotherapeutic drugs, and fluid reagents were added into 96-well culture plates. The cell density was 1000-3000 cells/well. Culture plates were incubated in standard MEM for 24 hours in a humidified medium at 37°C and 5% CO_2_.

About 100μL medium without serum was used to wash the cells two times. Further, standard medium (200μL), the single agent either DOXO (10μM or 100μM) or TMZ (10μM or 100μM), and their combination (10μM each) were added as treatments. Each experiment was conducted three times under the same conditions.

Proliferation assay

MTT assay (Roche Diagnostic GmbH, Basel, Switzerland) was used to determine the cytotoxic effect of drug monotherapy and combination therapy on both types of glioma cell lines (AC1B and GB1B). Six duplicates were produced with cells seeded by using 96-well culture plates with a density of 3000 cells/well in 200μL medium.

After each mono- or combination therapy, 10μL 3-[4,5-dimethylthiazol-2-yl]-2,5 diphenyl tetrazolium bromide (MTT) solution was added to each well and incubated at 37°C for four hours until purple crystals of formazan were obtained.

The assay is based on the principle that only metabolically active cells are able to cleave the yellow tetrazolium salt MTT and form the purple precipitate.

Cell lysis was induced with 100μL solubilization buffer and a spectrophotometer was utilized to measure the optical density (OD).

Statistical analysis

Microsoft Excel Student’s t-test with two-tailed distribution was utilized for statistical analysis of the cell viability (two-group comparison) to examine the variations across research groups. Only p < 0.05 results were considered statistically relevant.

Interactions between drugs (I) were classified by the multiplicative method as follows: additive (ADD) effect occurs when I1, 2 = I1 + I2; synergistic (SYN) effect occurs when I1, 2 > I1 + I2; and antagonistic or subadditive (SUB) effect occurs when I1, 2 < I1 + I2 [27] with I1, 2 being the observed survival while I1 + I2 is considered the predicted survival based on the cytotoxic effect of the two agents.

Results were reported as mean ± standard deviation (SD).

Each study was reproduced three times under the same experimental conditions.

## Results

DOXO treatment exhibits a more pronounced cytotoxic effect on high-grade glioma compared to low-grade glioma cells

This section of the study examines the cytotoxic effects of DOXO on two types of glioma cell lines: an astrocytoma cell line AC1B (a low-grade glioma) and a glioblastoma cell line GB1B (a high-grade glioma), using two different drug concentrations (10μM and 100μM).

The cell viability was measured on the 7th, 10th, and 14th day after agent administration.

**Figure 1:**
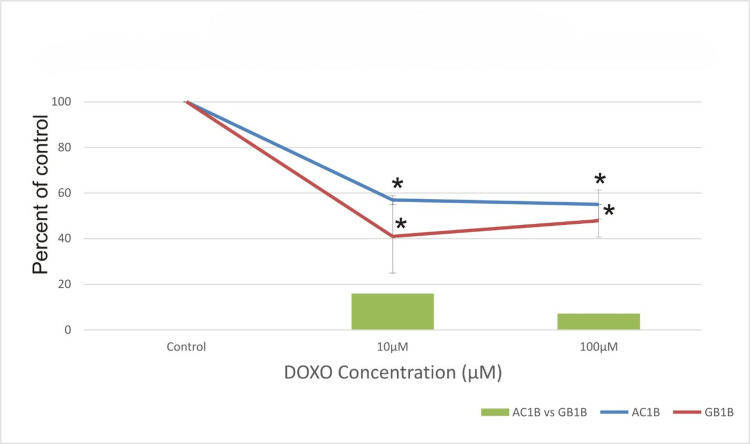
The effect of doxorubicin (DOXO) on the viability of two glioma cell lines: astrocytoma cells (AC1B cell line) and glioblastoma (GB1B cell line) after a seven-day course of treatment. Cells were harvested in standard medium and permitted to reach up to 70% confluence. Afterwards, cells were transferred onto multi-well plates and administered doses of 10μM and 100μM DOXO, respectively. The resulting cytotoxic effect is summarized as a percentage of control. A mean value was determined based on the results of three experiments performed under identical experimental conditions. Bars represent the difference in cell viability between AC1B and GB1B. * represents statistically significant values (p < 0.05) DOXO: doxorubicin

At seven days after DOXO treatment, both concentrations (10μM DOXO and 100μM DOXO) had a better effect on the GB1B cell line compared to AC1B, depicting 16% more cell death at 10μM DOXO and 7% at 100μM DOXO in high-grade glioblastoma than in low-grade astrocytoma (Figure 1).

A 43% death rate at 10μM DOXO and a 45% death rate at 100μM DOXO were found over the first seven days of treatment in the AC1B cell line, as seen in Figure 1, while for the GB1B cell line, the cell death rate was approximately 60% at 10μM DOXO and 53% at 100μM DOXO.

**Figure 2:**
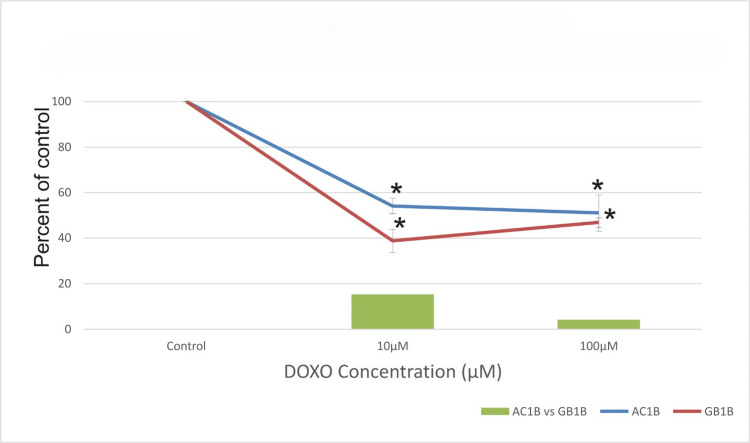
The effect of doxorubicin (DOXO) on the viability of two glioma cell lines: astrocytoma cells (AC1B cell line) and glioblastoma (GB1B cell line) after a 10-day course of treatment. Cells were harvested in standard medium and allowed to grow up to 70% confluence. Afterwards, cells were transferred onto multi-well plates and administered doses of 10μM and 100μM DOXO, respectively. The resulting cytotoxic effect is summarized as a percentage of control. A mean value was determined based on the results of three experiments performed under identical experimental conditions. Bars represent the difference in cell viability between AC1B and GB1B. * represents statistically significant values (p < 0.05) DOXO: doxorubicin

**Figure 3:**
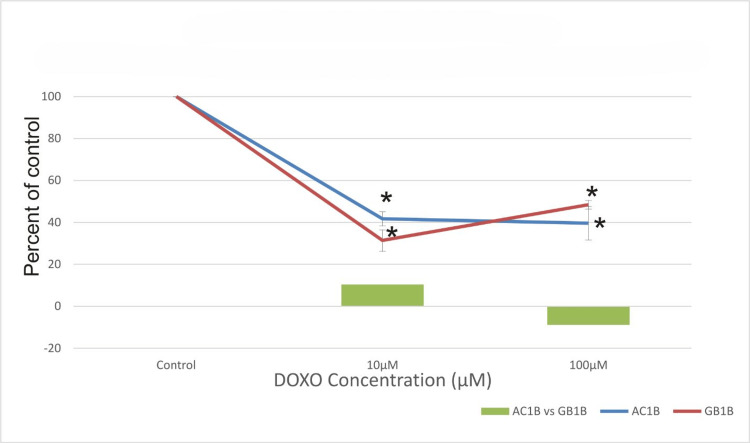
The effect of doxorubicin (DOXO) on the viability of two glioma cell lines: astrocytoma cells (AC1B cell line) and glioblastoma (GB1B cell line) after a 14-day course of treatment. Cells were harvested in standard medium and allowed to grow up to 70% confluence. Afterwards, cells were transferred onto multi-well plates and administered doses of 10μM and 100μM DOXO, respectively. The resulting cytotoxic effect is summarized as a percentage of control. A mean value was determined based on the results of three experiments performed under identical experimental conditions. Bars represent the difference in cell viability between AC1B and GB1B. * represents statistically significant values (p < 0.05) DOXO: doxorubicin

Figure 2 shows that 10 days after treatment, the viability of AC1B cells continued to decline with increasing concentration, with a cell death rate of 45% at 10μM and 49% at 100μM. However, for the GB1B cell line, the cytotoxic activity did not significantly increase compared to seven days after the treatment. The difference in response to the treatment with 10μM DOXO becomes smaller, and at 100μM the response of the two cell lines is essentially equal. As expected, the highest cytotoxic effect on AC1B has been determined at the 14th-day time mark, with 59% for 10μM and 61% for 100μM as observed in Figure 3. It can be observed that the cell viability of AC1B decreases in a dose-dependent manner with the concentration of the DOXO and with the time exposure (Figures 1-3). At 14 days, GB1B becomes much more resistant to the treatment (100μM DOXO), and the glioblastoma cell line acquires treatment resistance quickly. The drug's greatest impact was shown 14 days following therapy when a cell death rate of about 69% was observed (Figure 3). It is worth noting that for the 7th-day and 10th-day time mark, the cytotoxic activity of both treatments is better on GB1B (Figures 1, 2), but over a longer period, the high-grade glioma cell line demonstrates increased resistance, with a more adapted mechanism of survival (48% survival for GB1B compared with 39% survival for AC1B) (Figure 3).

TMZ treatment exhibits a more pronounced cytotoxic effect on low-grade glioma compared to high-grade glioma cells

**Figure 4:**
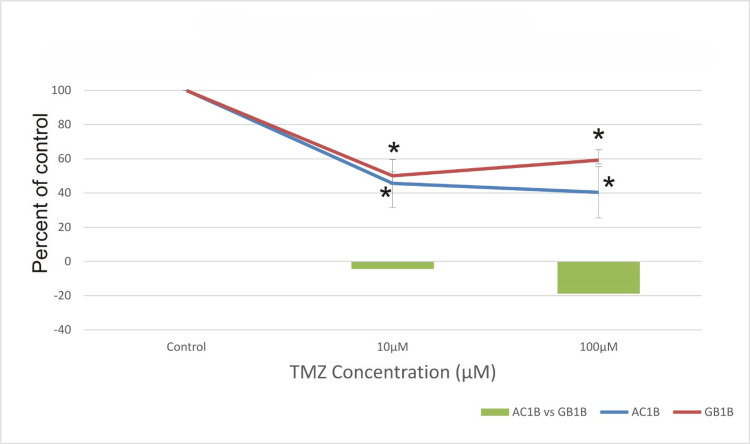
The effect of temozolomide (TMZ) on astrocytoma cells (AC1B cell line) and on glioblastoma cells (GB1B cell line) viability after a seven-day course of treatment. Cells were harvested in standard medium and allowed to grow up to 70% confluence. Afterwards, cells were transferred onto multi-well plates and administered doses of 10μM and 100μM TMZ, respectively. The resulting cytotoxic effect is summarized as a percentage of control. A mean value was determined based on the results of three experiments performed under identical experimental conditions. Bars represent the difference in cell viability between AC1B and GB1B. * represents statistically significant values (p < 0.05) TMZ: temozolomide

**Figure 5:**
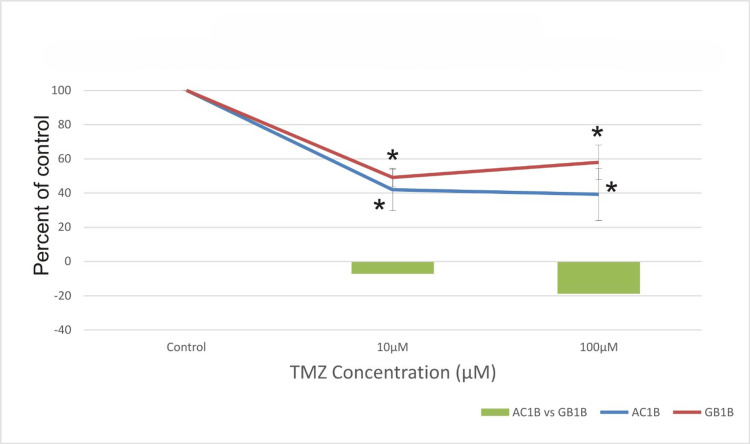
The effect of temozolomide (TMZ) on astrocytoma cells (AC1B cell line) and on glioblastoma cells (GB1B cell line) viability after a 10-day course of treatment. Cells were harvested in standard medium and allowed to grow up to 70% confluence. Afterwards, cells were transferred onto multi-well plates and administered doses of 10μM and 100μM TMZ, respectively. The resulting cytotoxic effect is summarized as a percentage of control. A mean value was determined based on the results of three experiments performed under identical experimental conditions. Bars represent the difference in cell viability between AC1B and GB1B. * represents statistically significant values (p < 0.05) TMZ: temozolomide

**Figure 6:**
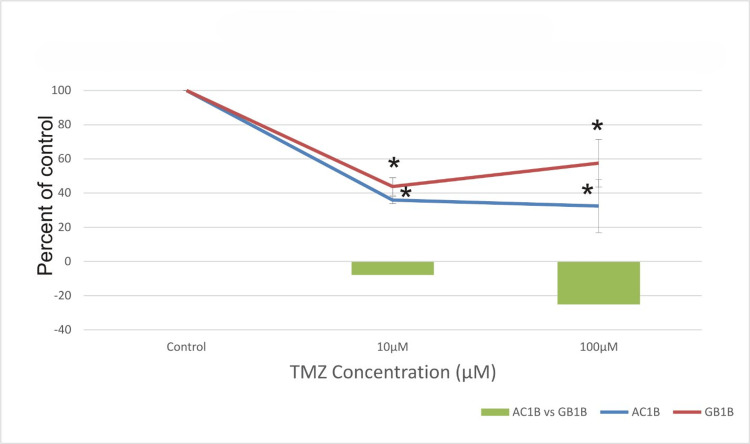
The effect of temozolomide (TMZ) on astrocytoma cells (AC1B cell line) and on glioblastoma cells (GB1B cell line) viability after a 14-day course of treatment. Cells were harvested in standard medium and allowed to grow up to 70% confluence. Afterwards, cells were transferred onto multi-well plates and administered doses of 10μM and 100μM TMZ, respectively. The resulting cytotoxic effect is summarized as a percentage of control. A mean value was determined based on the results of three experiments performed under identical experimental conditions. Bars represent the difference in cell viability between AC1B and GB1B. * represents statistically significant values (p < 0.05) TMZ: temozolomide

AC1B exhibited more sensitivity to TMZ treatment (under both concentrations - 10μM and 100μM) than GB1B, while TMZ resistance increased over time for the glioblastoma cells. The viability rate of astrocytoma cells was found to decline progressively in response to TMZ therapy, depending on the drug dosage and time exposure. As seen in Figures 4-6, the highest concentrations of TMZ had a better effect on AC1B, while at a lower dosage, the difference cannot be easily observed when the two cell lines are compared. As expected, the astrocytoma cell line (low-grade glioma) is more sensitive to the cytotoxic drug, compared to the high-grade glioma (glioblastoma cell line GB1B). For all concentrations of TMZ, the glioblastoma cell line was more resistant than the astrocytoma cell line.

It can be observed that seven days post-administration, the proportion of surviving cells was 45% for AC1B and 50% for GB1B at a concentration of 10μM and 40% for AC1B and 60% for GB1B at a concentration of 100μM (Figure 4).

Cell viability in the AC1B cell line decreased by 3% after a 10-day course of treatment with a concentration of 10μM TMZ and by 1% at 100μM TMZ compared to the 100μM TMZ treatment at the 7th-day mark (Figures 4, 5). Additionally, only a 1% difference was observed in the GB1B cell line after 10 days of TMZ treatment compared to seven days (Figures 4, 5), but cell survival was lower in AC1B than in GB1B cell lines.

As expected, the highest cytotoxic effect was obtained 14 days after treatment in both cell lines, with cell viability decreasing approximately by 68% at 100μM TMZ in the AC1B cell line and by only 43% when the 100μM TMZ was applied to the GB1B cell line (Figure 6).

Evaluation of the cytotoxic effect of TMZ and DOXO combined treatment on both low- and high-grade glioma cells

The combined cytotoxic properties of a low dose of DOXO and TMZ were analyzed compared to the single drug administration using a low-grade AC1B astrocytoma cell line and GB1B high-grade glioblastoma cell line.

**Figure 7:**
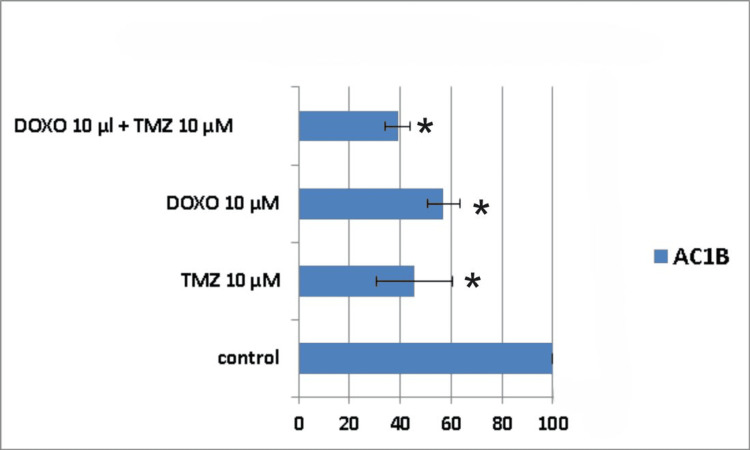
The impact of the combined treatment on astrocytoma cells (AC1B cell line) viability after a seven-day course of treatment. Cells were harvested in standard medium and allowed to grow up to 70% confluence. Afterwards, cells were transferred onto multi-well plates and treated with 10μM doxorubicin (DOXO), 10μM temozolomide (TMZ), and the combination of low doses of the two chemotherapeutic drugs (10μM TMZ+10μM DOXO). The resulting cytotoxic effect is summarized as a percentage of control. A mean value was determined based on the results of three experiments performed under identical experimental conditions. * represents statistically significant values (p < 0.05) DOXO: doxorubicin; TMZ: temozolomide

Seven days following treatment, cell death was around 61% for the combined treatment group (10μM TMZ + 10μM DOXO) in the AC1B cell line, whereas for the single drug administration groups, cell death was 44% for 10μM DOXO and 55% for 10μM TMZ, respectively (Figure 7).

**Figure 8:**
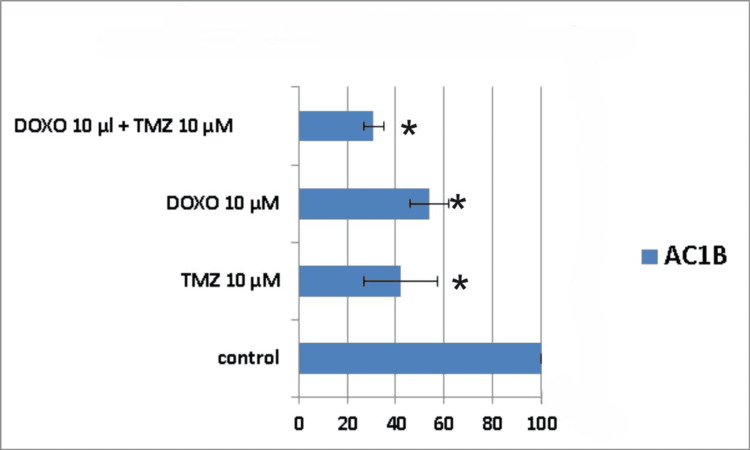
The impact of the combined treatment on astrocytoma cells (AC1B cell line) viability after a 10-day course of treatment. Cells were harvested in standard medium and allowed to grow up to 70% confluence. Afterwards, cells were transferred onto multi-well plates and treated with 10μM doxorubicin (DOXO), 10μM temozolomide (TMZ), and the combination of low doses of the two chemotherapeutic drugs (10μM TMZ+10μM DOXO). The resulting cytotoxic effect is summarized as a percentage of control. A mean value was determined based on the results of three experiments performed under identical experimental conditions. * represents statistically significant values (p < 0.05) DOXO: doxorubicin; TMZ: temozolomide

Ten days post-treatment, the cytotoxic effect was 58% for 10μM TMZ, 45% for 10μM DOXO, and 70% for 10μM TMZ+10μM DOXO (Figure 8).

**Figure 9:**
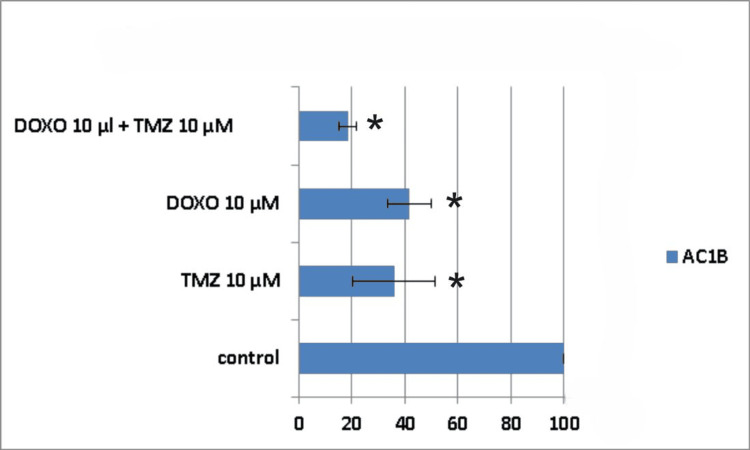
The impact of the combined treatment on astrocytoma cells (AC1B cell line) viability after a 14-day course of treatment. Cells were harvested in standard medium and allowed to grow up to 70% confluence. Afterwards, cells were transferred onto multi-well plates and treated with 10μM doxorubicin (DOXO), 10μM temozolomide (TMZ), and the combination of low doses of the two chemotherapeutic drugs (10μM TMZ+10μM DOXO). The resulting cytotoxic effect is summarized as a percentage of control. A mean value was determined based on the results of three experiments performed under identical experimental conditions. * represents statistically significant values (p < 0.05) DOXO: doxorubicin; TMZ: temozolomide

The peak cytotoxic effect was observed at the 14th-day time mark, but with no synergism present (Table 1). A 10μM TMZ dose induced death in 65% of the astrocytoma cells, a 10μM DOXO dose produced 59% cell death, and their combination led to a cell viability of only 18% (Figure 9).

**Table 1:** The interaction between combined treatment in astrocytoma cells (AC1B cells). DOXO: doxorubicin; TMZ: temozolomide

Time (days)	TMZ (μM)	DOXO (μM)	Predicted survival	Observed survival	Effect
7	10	10	0.25	0.39	Subadditive
10	10	10	0.22	0.31	Subadditive
14	10	10	0.12	0.18	Subadditive

Throughout the assessed time frames, it can be noticed that all types of treatments displayed a continuous decrease in AC1B cell viability, but with a difference between combination therapy and DOXO treatment of 21% and between the dual treatment combination and TMZ treatment of 14%.

**Figure 10:**
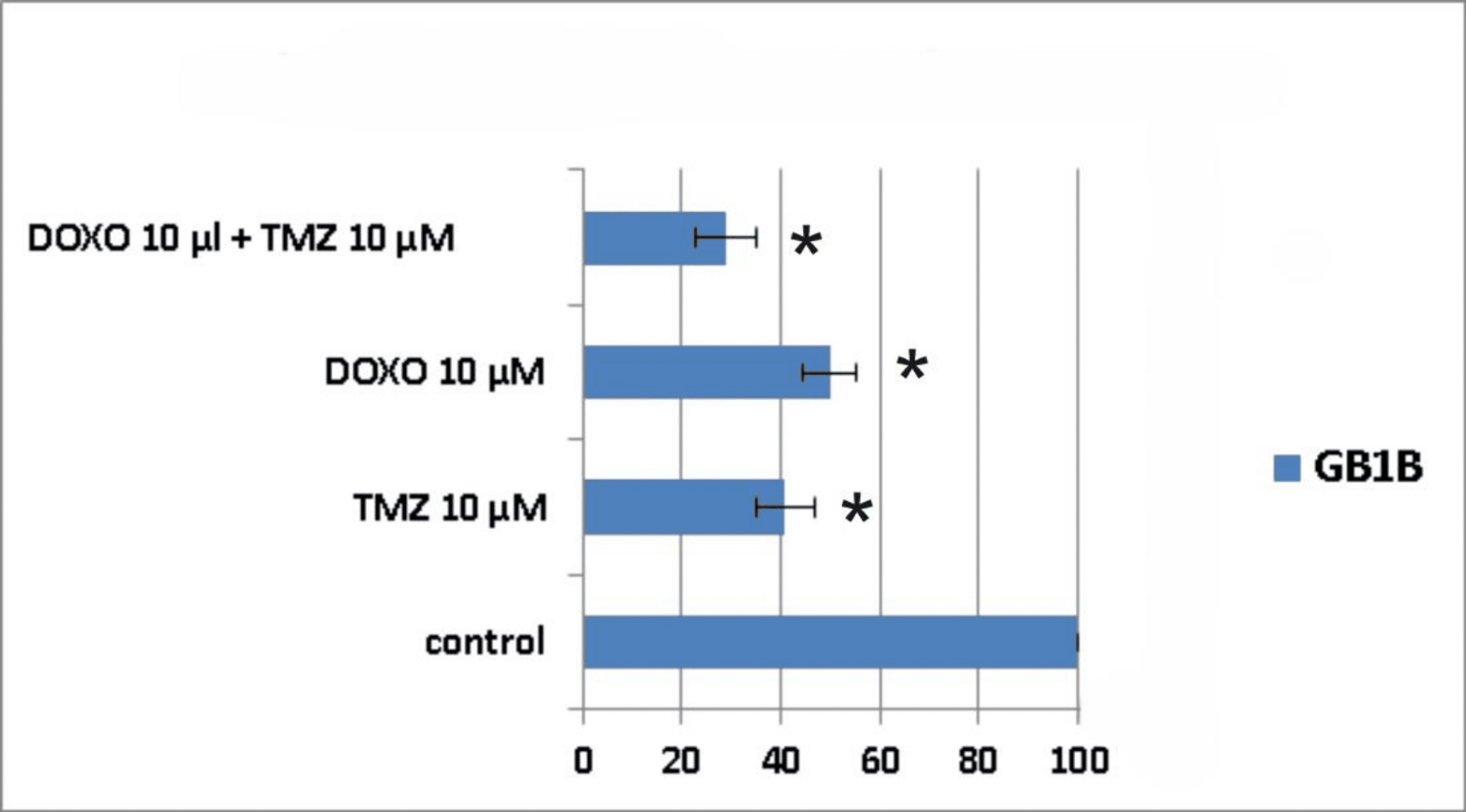
The impact of the combined treatment on glioblastoma cells (GB1B cell line) viability after a seven-day course of treatment. Cells were harvested in standard medium and allowed to grow up to 70% confluence. Afterwards, cells were transferred onto multi-well plates and treated with 10μM doxorubicin (DOXO), 10μM temozolomide (TMZ), and the combination of low doses of the two chemotherapeutic drugs (10μM TMZ+10μM DOXO). The resulting cytotoxic effect is summarized as a percentage of control. A mean value was determined based on the results of three experiments performed under identical experimental conditions. * represents statistically significant values (p < 0.05) DOXO: doxorubicin; TMZ: temozolomide

When the treatment was applied to the GB1B cell line, the results showed that seven days after administration, the cytotoxic effect was 72% for the combined treatment group (TMZ 10μM + DOXO 10μM), whereas for the single drug administration, cell death was 60% for DOXO 10μM and 50% for TMZ 10μM, respectively (Figure 10).

**Figure 11:**
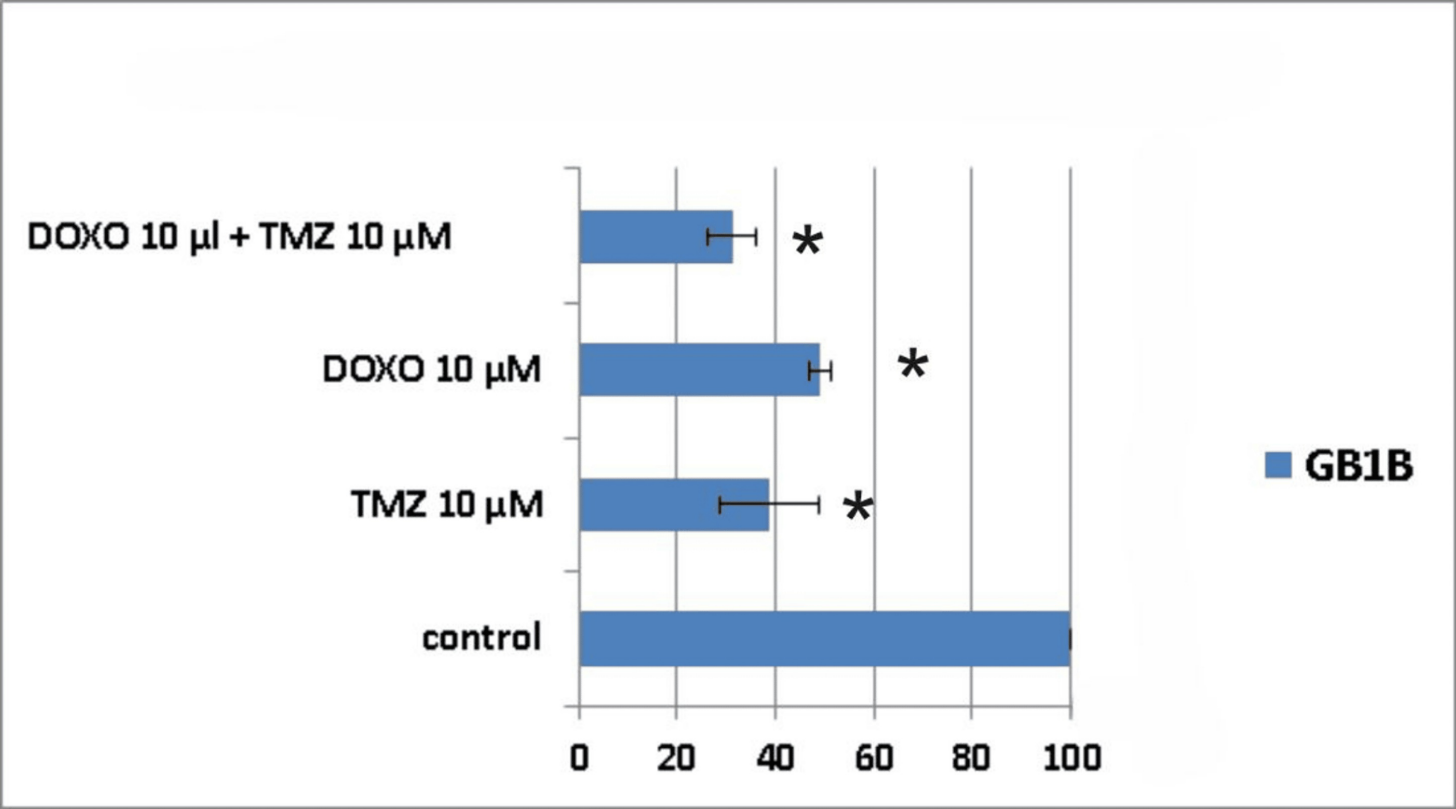
The impact of the combined treatment on glioblastoma cells (GB1B cell line) viability after a 10-day course of treatment. Cells were harvested in standard medium and allowed to grow up to 70% confluence. Afterwards, cells were transferred onto multi-well plates and treated with 10μM doxorubicin (DOXO), 10μM temozolomide (TMZ), and the combination of low doses of the two chemotherapeutic drugs (10μM TMZ+10μM DOXO). The resulting cytotoxic effect is summarized as a percentage of control. A mean value was determined based on the results of three experiments performed under identical experimental conditions. * represents statistically significant values (p < 0.05) DOXO: doxorubicin; TMZ: temozolomide

Ten days post-treatment, the cytotoxic effect was 51% for TMZ 10μM, 62% for DOXO 10μM, and 69% for TMZ 10μM + DOXO 10μM combination (Figure 11).

**Figure 12:**
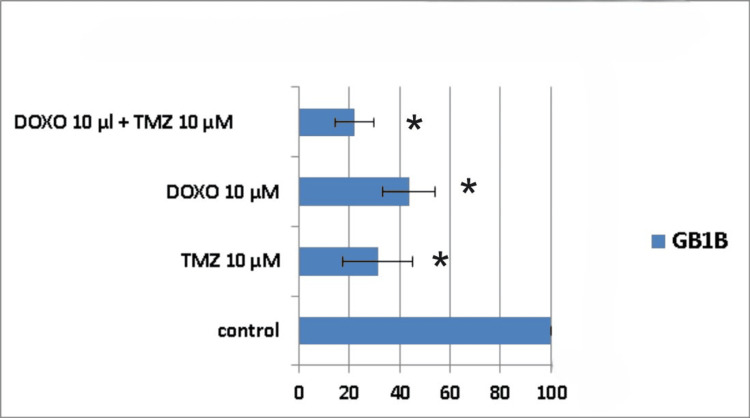
The impact of the combined treatment on glioblastoma cells (GB1B cell line) viability after a 14-day course of treatment. Cells were harvested in standard medium and allowed to grow up to 70% confluence. Afterwards, cells were transferred onto multi-well plates and treated with 10μM doxorubicin (DOXO), 10μM temozolomide (TMZ), and the combination of low doses of the two chemotherapeutic drugs (10μM TMZ+10μM DOXO). The resulting cytotoxic effect is summarized as a percentage of control. A mean value was determined based on the results of three experiments performed under identical experimental conditions. * represents statistically significant values (p < 0.05) DOXO: doxorubicin; TMZ: temozolomide

As for the AC1B cell line and also for the GB1B cell line, the maximum cytotoxic effect occurred on the 14th-day mark (Figure 12), but there was no evidence of synergism (Table 2). The 10μM TMZ induced death in 57% of the glioblastoma cells, the 10μM DOXO achieved 69% cell death, and their combination led to a maximum cytotoxic effect of 78% (Figure 12). Therefore, it can be noticed that all types of treatments displayed a continuous decrease in GB1B cell viability.

**Table 2:** The interaction between combined treatment in glioblastoma cells (GB1B cells). DOXO: doxorubicin; TMZ: temozolomide

Time (days)	TMZ (μM)	DOXO (μM)	Predicted survival	Observed survival	Effect
7	10	10	0.2	0.29	Subadditive
10	10	10	0.19	0.31	Subadditive
14	10	10	0.14	0.22	Subadditive

Moreover, TMZ and DOXO concurrent treatment interactions were calculated to compare the efficacy of the dual therapy with the single treatment.

None of the combinations used in this study on the AC1B cells proved a synergic or additive activity, as seen in Table 1.

TMZ and DOXO concurrent treatment interactions were also calculated for the GB1B cell line to compare dual treatment with single therapy. Similar to the AC1B cell line, none of the combined approaches used in the study demonstrated synergistic or additive activity in the G1B1 cell line (Table 2).

## Discussion

Low-grade gliomas grow slowly, but if left untreated, they can progress to high-grade gliomas. Rapid diagnosis and treatment are necessary to prevent increased morbidity and mortality. However, treatment can be very effective for low-grade glioma, but currently, the management of high-grade glioma is very difficult to achieve.

Single-agent therapy has been shown to be less effective compared to combination therapy; therefore, one of the potential strategies to treat low-grade tumors is combination therapy. It has not yet been established how effective different approaches are compared to RT alone, TMZ alone, or combined therapy in gliomas [9]. Monotherapy with TMZ was associated with a shorter PFS than RT alone in high-risk WHO Grade 2 astrocytomas [10]. Instead, similar results with single-agent TMZ chemotherapy versus RT have been presented in Grade 3 gliomas [28].

Additionally, having a different mechanism of action than TMZ, DOXO is another very effective chemotherapeutic drug used in a variety of malignancies.

Recently published data showed that TMZ and DOXO co-administration generates better results than the therapy with TMZ alone. Nevertheless, in terms of long-term survival, their combination did not prove the expected increased effects compared to the therapy with DOXO [29].

In our previous studies, we proved that the GB2B and GB8B cell lines were sensitive to both DOXO and TMZ, exhibiting increased cytotoxicity when treated with 10μM and 100μM TMZ at 7, 10, and 14 days post-administration, and with similar effects when treated with the same concentrations of DOXO [24,25].

The current study was undertaken to compare the effect of the single and combined treatment of TMZ and DOXO in a low-grade glioma - an astrocytoma cell line AC1B and a high-grade glioma - a glioblastoma GB1B cell line. Results showed that single and combined therapy induced a cytotoxic effect in both cell lines, with a more pronounced cell death rate in high-grade glioma when treated with DOXO in monotherapy in comparison to the AC1B cell line. The astrocytoma cell line presented a continuous increase of the cytotoxic effects throughout the entire treatment regimen, while the glioblastoma cell line presented a better response to the DOXO single therapy and a continuous decrease in cell viability, but with a rapid increase in drug resistance at the end of the assessed time frames, especially in the TMZ single therapy group. When it comes to the DOXO single therapy groups, it can be observed that the greatest difference in response is at 10μM with a better response in GB1B (cell death rate of 69% in GB1B cell line versus 59% in AC1B at 14 days), while at 100μM, the response of the two cell lines was essentially equal at 10 days. At 14 days, GB1B became much more resistant to the 100μM DOXO treatment, while also acquiring resistance very quickly to the treatment. The AC1B cell line was more sensitive to TMZ treatment than the GB1B cell line, and GB cells’ resistance to TMZ increased over time, especially at 100μM TMZ. TMZ and DOXO combined treatment did not exert a synergistic cytotoxic effect on both low- and high-grade glioma cells, with a maximum cytotoxicity of 82% in the AC1B cell line at 14 days.

Zhang et al. have studied in an in vitro experiment the cytotoxic effect of TMZ and DOXO on glioblastoma cell lines, where they found a synergistic effect on the U251MG cell line [30]. Our study did not exhibit a synergistic effect on the low-passage cell lines established from the patient’s specimens. These results may be attributed to the low-passage cell lines that maintain, to a certain degree, the tumor’s characteristics from which they were derived in comparison to the high-passage cell lines that are prone to genetic changes so that they no longer exhibit the same characteristic as the tumor they were derived from. Therefore, in vitro studies may not be accurately reproduced in an in vivo setting, where additional factors contribute to the development of treatment resistance and sensitivity to certain pharmacological agents.

Study limitations

The results of our research are based on a limited number of glioma cell lines. Since the behavior of the original tumor may not be fully replicated in vitro, this study’s results can be limited and may not be regarded in a general manner. Other factors present in an in vivo setting, such as the tumor microenvironment, BBB, brain-tumor barrier, immunological response, and interactions between tumor and non-tumor cells, contribute to a complex and varied clinical outcome and prognosis. Thus, the concentrations of DOXO and TMZ may not reflect a genuine in vivo pharmacokinetic profile due to the variability of drug penetration and metabolism impacted by the in vivo factors.

Although our study offers valuable insights into the cytotoxic effects of DOXO and TMZ on both low-grade and high-grade glioma cells, the current study was specifically focused on the short-term and immediate cytotoxic effects. Longer-term effects and drug toxicity or resistance impose further studies, such as animal models.

## Conclusions

The current study evaluated the cytotoxic effects of DOXO and TMZ on two glioma cell lines: AC1B (grade 2 astrocytoma) and GB1B (glioblastoma). Both medications were used in two concentrations (10μM and 100μM) and administered in monotherapy and combination therapy. Their effects were observed over a period of 7, 10, and 14 days. Taken together, we concluded that DOXO and TMZ single and dual treatments exhibited cytotoxic effects, but with subadditive values. Nevertheless, a more pronounced cytotoxic effect on high-grade glioma cells compared to low-grade glioma cells was observed in the DOXO treatment groups at 10μM and a better response in overall treatment with TMZ in low-grade glioma cells, while high-grade glioma cells became more resistant to the treatments. However, the combined treatment on both cell lines failed to synergistically induce cell death.
